# Emerging role of glucocorticoid receptor in castration resistant prostate cancer: A potential therapeutic target

**DOI:** 10.7150/jca.32497

**Published:** 2020-01-01

**Authors:** Raj Kumar

**Affiliations:** Department of Biomedical Sciences, College of Medicine, University of Houston, Houston, TX, USA.

**Keywords:** castration resistant prostate cancer, glucocorticoid receptor, androgen receptor, therapeutic target

## Abstract

Glucocorticoids are used as co-medication with chemotherapy for solid tumors to reduce inflammation as well as cytotoxic side effects and are effective in easing symptoms related to chemotherapy. However, emerging evidence suggests that glucocorticoids may contribute to failure of chemotherapy and tumor progression of castration resistant prostate cancer (CRPC). Thus, in recent years, glucocorticoid signaling pathway has become an important therapeutic target for CRPC. Understanding the exact mechanism of GR actions in CRPC is still work in progress. There are studies suggesting that GR expression can be upregulated following antiandrogen therapy and can contribute to resistance to hormone therapies. Therefore, attempts are being made to develop selective glucocorticoid receptor modulators that specifically antagonize GR activity in CRPC, and thereby provide clinical benefit by blocking the GR mechanism for tumor growth. However, more targeted approaches are needed to understand the role of the GR-mediated target gene expressions in the CRPC that could in near future lead to better therapeutic options for patients with CRPC. This review highlights current perspectives on the actions of glucocorticoids during tumor progression and metastasis of CRPC.

## Introduction

Glucocorticoids, secreted from the adrenal gland, are necessary for human life and regulate various physiological processes to maintain homeostasis 1, 2. The glucocorticoids are responsible for modulating essential metabolic, cardiovascular, immune, and behavioral functions 3, 4. Because of their powerful anti-inflammatory and immunosuppressive actions, synthetic glucocorticoids are one of the most widely prescribed drugs in the world today and are used for treating inflammatory and autoimmune diseases, such as asthma, allergy, sepsis, rheumatoid arthritis, ulcerative colitis, and multiple sclerosis 5, 6. They are also commonly prescribed to prevent organ transplant rejection and to treat cancers of the lymphoid system such as leukemias, lymphomas, and myelomas 7, 8. However, patients chronically treated with synthetic glucocorticoids are prone to severe side effects including osteoporosis, diabetes, obesity, glaucoma, growth retardation in children, and hypertension among others 9-11.

In cancer therapies, glucocorticoids have not only been widely used in the treatment of lymphoid malignancies to induce cell apoptosis, but also as co-medication with chemotherapy for solid tumors to reduce inflammation as well as cytotoxic side effects 12, 13. In many types of solid tumors, co-treatment with glucocorticoids is effective in easing symptoms related to chemotherapy or cancer per se 12, 13. However, glucocorticoid therapy may also increase the risk for failure of chemotherapy 13-16. Emerging evidence suggests that glucocorticoids may contribute to failure of chemotherapy and tumor progression of many types of solid tumors including triple negative breast cancer (TNBC) and castration resistant prostate cancer (CRPC) 14-19. Interestingly, glucocorticoids appear to slow cell proliferation in estrogen receptor-positive breast cancer whereas in TNBC, glucocorticoids inhibit chemotherapy-induced cytotoxicity by preventing apoptosis, resulting into increased cell proliferation 16, 17. Similarly, in case of androgen-dependent prostate cancer, glucocorticoids appear to slow proliferation of tumor cells whereas in CRPC, glucocorticoids act quite differently, leading to tumor progression 18, 19.

It is amazing how glucocorticoids use different mechanisms depending on various cancer types and specific biological targets to promote or inhibit cancer progression and proliferation 20, 21. The role of glucocorticoids in the treatment of some cancers such leukemias and lymphomas is reasonably well understood 22, 23. However, the underlying mechanisms of the pro-tumorigenic effects of glucocorticoids in solid tumors are not well known. In recent years, though, pro-tumorigenic roles of glucocorticoids in the CRPC has emerged and glucocorticoid signaling pathway has become an important therapeutic target for these cancer types 18, 19. In this review, we are highlighting some of the latest findings and perspectives on the actions of glucocorticoids during tumor progression and metastasis of CRPC.

## Structure and functions of glucocorticoid receptor

**Figure 1:**
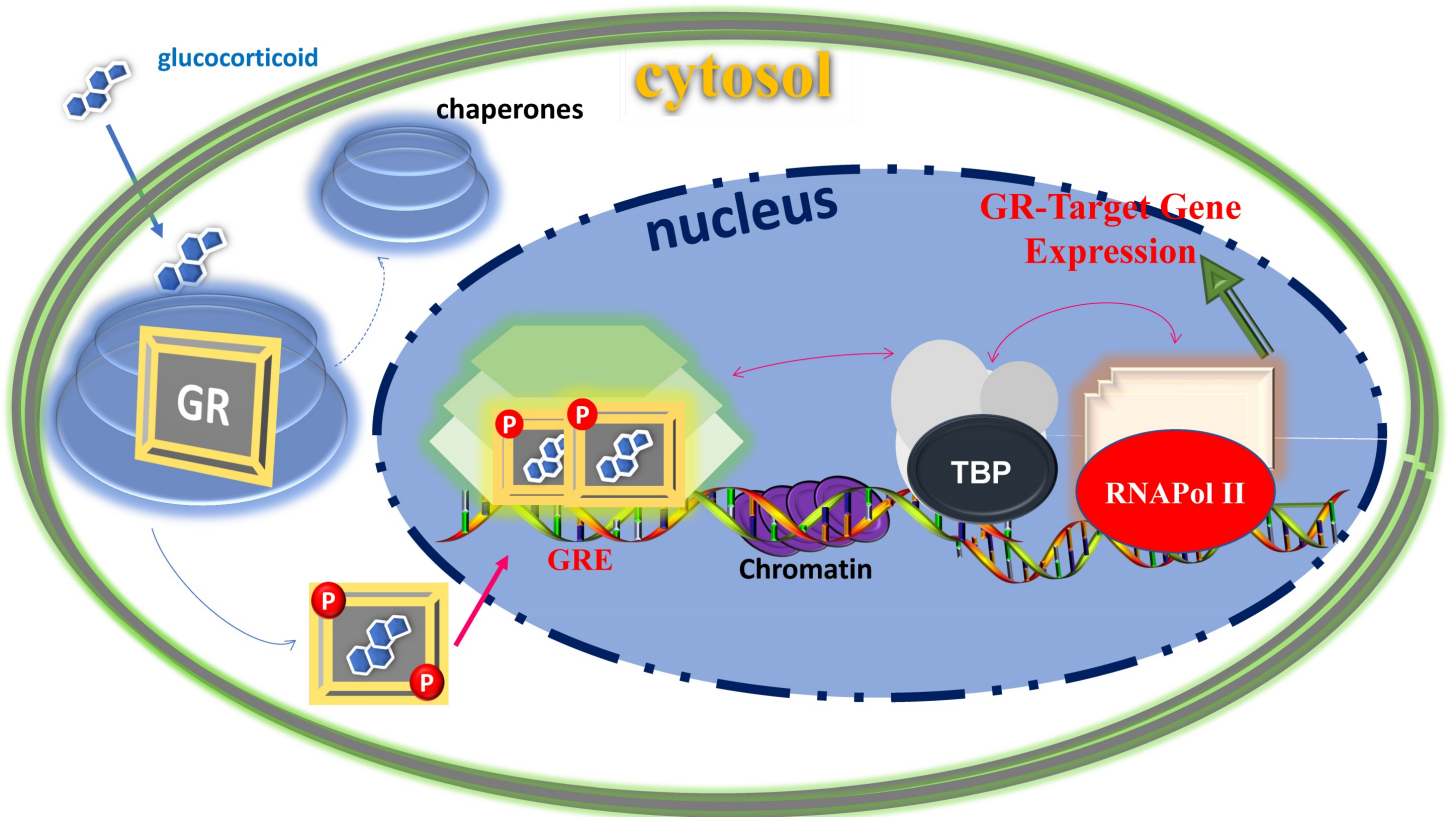
** Classical action of gene regulation by the GR-mediated glucocorticoid signaling.** Unliganded receptor is located in the cytosol associated with several heat shock and other chaperone proteins (shown by circular shades around GR). Ligand binding dissociates GR from these associated proteins, and ligand bound, phosphorylated GR translocates to the nucleus where it dimerizes and binds to site-specific DNA binding sequences and interacts with several other coregulatory proteins (shown by hexagonic shapes), remodels chromatin structure (shown by purple color in DNA sequences), and with certain mediators that allow cross talk between the GR and the basal transcription machinery including TATA-Box and RNA Pol II complexes (shown by arrows) affect transcription of GR-target gene. Based on 74, 75.

The physiological and pharmacological actions of glucocorticoids are mediated via the glucocorticoid receptor (GR) at the level of gene regulation 24, 25. The GR belongs to the superfamily of ligand- dependent intracellular transcription factors 26-28. Unliganded GR resides in the cytosol associated with various proteins including chaperones (e.g., hsp90, hsp70, and p23) and immunophilins of the FK506 family (e.g., FKBP51 and FKBP52) 29, 30. These proteins maintain the receptor in a conformation that is transcriptionally inactive but favors high affinity ligand binding 29, 30. Once ligand-bound, the GR undergoes conformational rearrangements resulting into dissociation of these proteins as well as exposing the nuclear localization signals to rapidly translocate into the nucleus where it induces or represses the transcription of its target genes by binding directly to its specific response element DNA sites and/or by physically interacting with other coregulatory proteins (Figure 1). For example, GR activation can induce apoptosis in lymphocytes 31, 32, whereas its activation results in inhibition of apoptosis in breast epithelial cells 33. The GR can also act through cross-talk with other transcription factors such as activator protein-1, signal transducers and activators of transcription-5 and nuclear factor-ĸB 24-28. In addition to the genomic mode of actions, mainly, via their transcriptional regulation of genes, increasing evidence suggests that glucocorticoids can also act through non-genomic signaling mechanism, which does not require nuclear translocation of GR and GR-mediated transcription 34, 35. These effects are thought to occur by the membrane-bound or cytoplasmic GR 34, 35. Although, the precise mechanisms of non-genomic glucocorticoids signaling are still under investigation and may provide novel therapeutic targets for related diseases in the future.

Consistent with the pleiotropic actions of glucocorticoids, GR is expressed in nearly every cell of the body and is necessary for life after birth 7. The transcriptional activity of GR varies widely between cell types, thus accounting for the diverse and sometimes opposite physiological effects of glucocorticoid in different tissues 7. Ligand bound GR also undergoes post translational modifications including phosphorylation and is tightly regulated through cell/tissue specific kinases and phosphatases 36-38. Phosphorylation affects GR stability, nuclear-cytoplasmic shuttling and its interactions with other transcriptional factors ultimately leading to different regulations of GR-responsive genes 36-39. Like other members of the steroid receptor family, GR protein consists of three major functional domains: an N-terminal domain (NTD), a central DNA-binding domain (DBD), and a C-terminal ligand binding domain (LBD) 24-28. The DBD and LBD are responsible for site-specific DNA binding and steroid/hormone binding, respectively 24-28. The NTD houses a powerful transcriptional activation function (AF1), which is constitutively active 24-28. The LBD also possesses a transcriptional activation function (AF2), which acts in a ligand-dependent manner 39. Both AF1 and AF2 are the major sites for the GR's interaction with various coregulatory proteins including the basal transcription machinery proteins 39. The complete action of the GR requires a synergistic effect of both AF1 and AF2 in a cell/tissue-dependent manner 40. Recent studies have shown that unlike DBD and LBD, which exist as globular proteins with well-defined 3-D structure 39, the NTD/AF1 exits as an intrinsically disordered protein (ID), commonly found in many transcription factors including other members of the steroid hormone receptor family 40-42. The NTD/AF1 is also the primary site for post-translational modifications, particularly all the functionally important known phosphorylation sites are located in this region 36-39. Due, in part, to the ID nature of the NTD/AF1, the full length 3-D structure determination of the GR as well as other members of the steroid hormone receptor family has been difficult so far 39. The emerging picture is that in order to access the entire GR signaling spectrum for the development of novel and potent therapeutic agents, we must determine the structure of not only individual domains but of full-length GR 39.

## Glucocorticoid receptor in castration resistant prostate cancer

Prostate cancer is second leading cause of cancer-related death among men in the USA 43. Since androgen receptor (AR) plays a critical role in the development and progression of prostate cancer, androgen deprivation therapy (ADT) with lowering of serum testosterone levels to castrate levels has been the mainstay of therapy for these patients for years 44-47. Standard approaches to ADT include surgical bilateral orchiectomy or medical orchiectomy using a gonadotropin releasing hormone (GnRH) agonist alone or in combination with an anti-androgen (antagonist) 47. Some recent studies suggest that surgical orchiectomy may have lower risk for complications and side effects than medical castration with GnRH agonists in treatment of newly diagnosed patients with metastatic prostate cancer 47.

Although ADT is palliative, it can normalize serum levels of prostate-specific antigen in majority of patients, they eventually experience disease progression despite treatment 48. Because early in their development, prostate cancers need relatively high levels of androgens to grow, ADT typically works well at this stage 47, 48. Such prostate cancers are commonly called androgen-sensitive or androgen-dependent 46. Over time, however, prostate cancer tends to relapse and progresses into an incurable state and becomes refractory to ADT 49, 50. These patients ultimately progress to castration resistance, wherein prostate cancer cells become resistant to ADT and develop mechanisms to proliferate despite castrate levels of testosterone 51, 52. This state of disease which continues to grow despite the undetectable levels of androgens is known as CRPC and is highly detrimental 49, 53.

**Figure 2:**
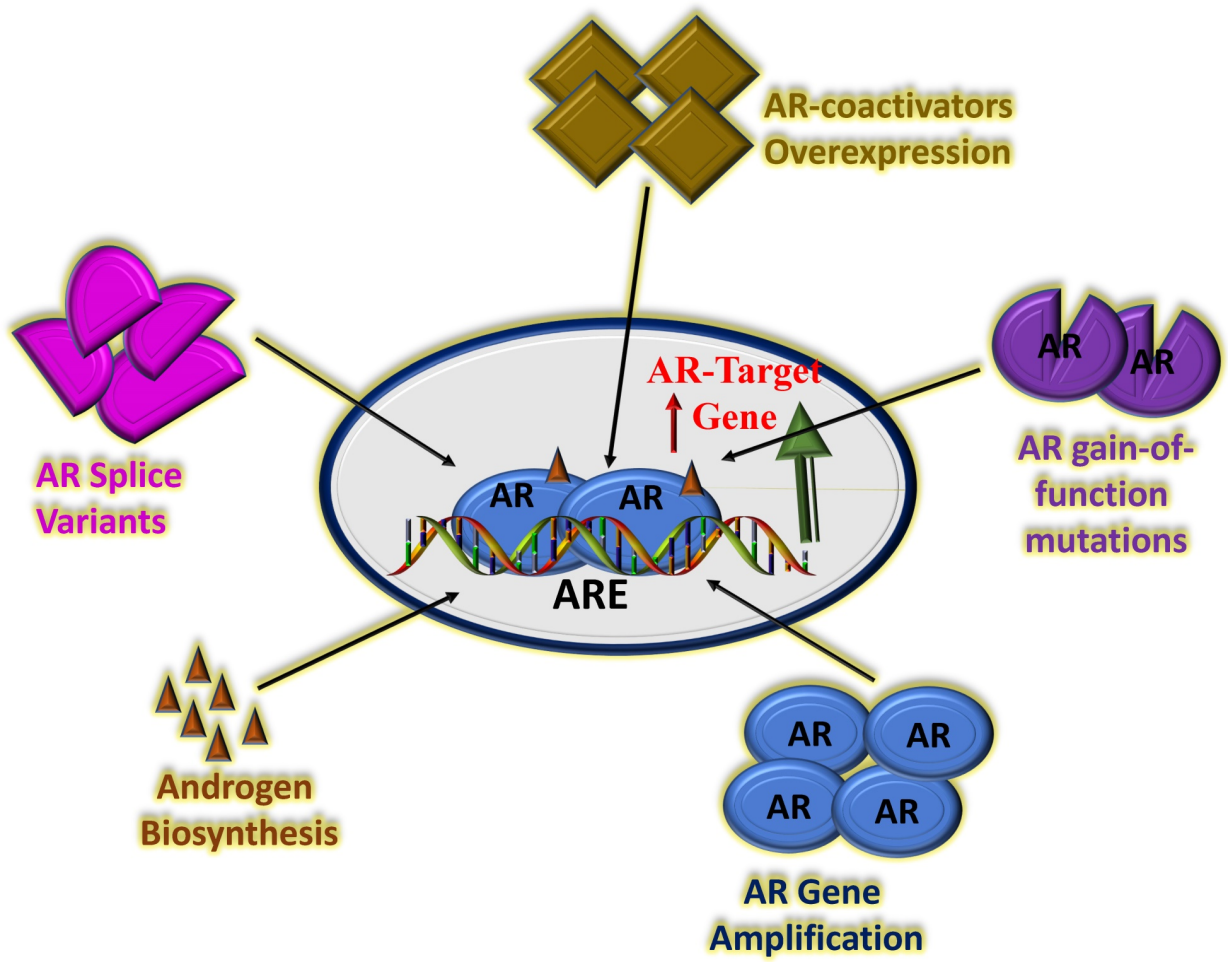
** Potential mechanisms of CRPC driven by continued AR transcriptional activity.** There are several possible factors that can affect AR activity including amplification of the AR gene leading to overexpression of AR protein, AR gain-of-function mutations, overexpression of AR co-activator proteins, increased adrenal androgen biosynthesis and/or AR splice variants with truncated LBD. Based on 66, 67, 76, 77.

Understanding the exact mechanism of CRPC is still work in progress. In recent years, AR amplification, AR splice variant expression, AR mutation and aberrant AR co-regulators activities have been shown to be involved in CRPC 54-56. Recently, the role of AR splice variant expression in the progression of CRPC has been studied extensively including the most predominant splice variant 7 (AR-V7 or AR3), which encode protein isoforms that activate AR pathway in the absence of androgens 57. Several second-generation AR signaling inhibitors such as cytochrome P450 17α-hydroxy/17,20-lyase (*CYP17*) inhibitor, abiraterone acetate have been successfully tested in patients with metastatic CRPC 58. Though, the second-generation AR antagonist, enzalutamide prolongs CRPC patient survival yet prostate cancer resistance to potent AR pathway blockade is inevitable 59, 60. Multiple mechanisms of resistance have been proposed including gain-of-function mutations in AR LBD, expression of constitutively active AR splice variants, and more recently increased expression and activity of GR, which can promote CRPC progression following AR blockade 61-64. There are suggestions that GR expression can be upregulated following antiandrogen therapy including enzalutamide and that GR upregulation can bypass the AR pathway and contribute to resistance to hormone therapies 65-67. These observations are important, and therefore warrant new insights into the mechanisms of actions of the GR in the drug resistance (Figure 2). A precise understanding of the mechanisms of action will immensely help in the development of next-generation therapies for CRPC with better clinical outcomes.

Efforts to block the effect of GR in CRPC using classical GR antagonist (RU486 or mifepristone) have been only partially successful 68. This may be due, in part, to the fact that mifepristone can also modulate AR signaling (though weakly) as well as alter the metabolism of other therapeutics through its potent effects on cytochrome P450 enzymatic activity 69, 70. Therefore, attempts are being made to develop selective glucocorticoid receptor modulators that specifically antagonize GR activity in CRPC (without significant binding to other members of the steroid hormone receptor family), and thereby provide clinical benefit by blocking the GR mechanism for tumor growth 71, 72. There are also clinical trials underway to test whether concomitant AR and GR antagonism using mifepristone and enzalutamide can increase the time to endocrine therapy resistance (Clinical Trials Entitled: “Enzalutamide and Mifepristone in Treating Patients With Metastatic Hormone Resistant Prostate Cancer”; Identifier: NCT 02012296). Despite these efforts, currently no FDA- approved therapies specific to enzalutamide-resistant CRPC are in clinical use. Therefore, GR could be a potential therapeutic target in this context. Due to the non-specific activity of mifepristone, development of highly specific GR modulators with principally GR antagonist activity have recently been tested under *in vitro* and *in vivo* studies. 73. In this study, two structurally distinct, yet highly selective GR modulators with principally GR antagonistic activity were found to selectively inhibit GR activity through the inhibition of the expression of genes associated with proliferation pathways 73.

## Summary and future perspectives

**Figure 3:**
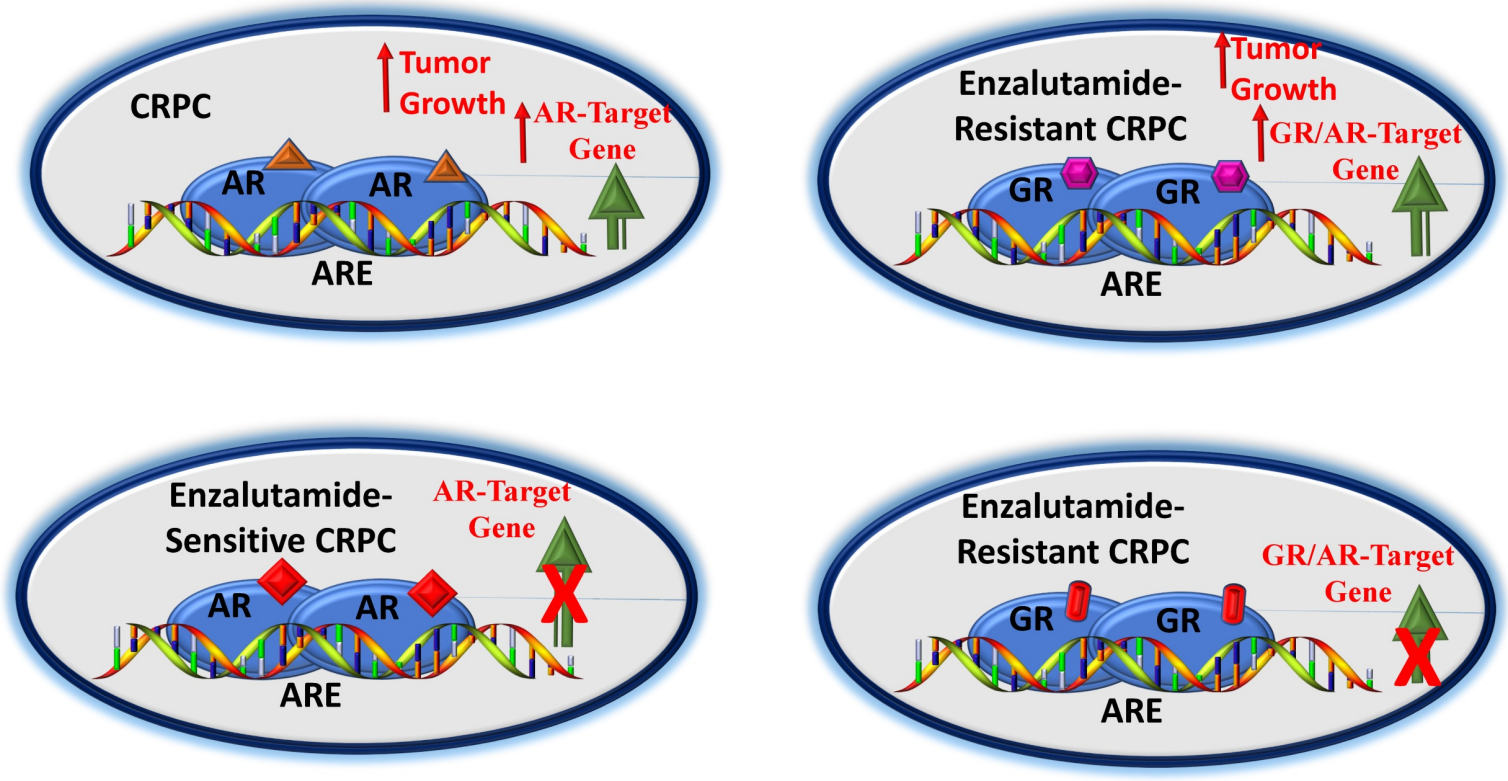
** A potential pathway by which GR affects CRPC.** In early stages of CRPC, an agonist-bound AR can upregulate AR-target genes leading to tumor growth (A). In this androgen-sensitive stage, blockade of AR by an antagonist (e.g., enzalutamide) can block AR-target genes (B). However, enzalutamide-resistance may result into higher GR expression, leading to activation of a subset of AR- and/or as GR- target genes causing tumor growth (C). GR antagonist may overcome GR-driven resistance to antiandrogens (D). Based on 65-67, 76, 77.

Generally, the standard treatment for men with metastatic prostate cancer involves either ADT alone or in combination with chemotherapy. A diagnosis of metastatic CRPC often means that the patient is no longer responding to ADT type of therapy. Under such circumstances, the available cancer treatment options are limited. In recent year, several pathways have been described for the progression and development of CRPC, which have led to the clinical utility of enzalutamide in the treatment of nonmetastatic CRPC. However, enzalutamide benefits are limited due to the development of drug resistance. Therefore, scientific community has been actively pursuing the mechanisms of this drug resistance. In last few years, a significant progress has been made to understanding the mechanisms of these effects that include expression of constitutively active AR- splice variants, point mutations, gene amplification and/or overexpression. More recently the role of higher expression and transcriptional activity of the GR in enzalutamide-resistant CRPC has caught an immense attention (Figure 3). Inhibition of the GR activity has been found to be effective under *in vitro* and *in vivo* conditions that has led to the development of small molecule selective GR modulators. However, more targeted approaches are needed to understand the role of the GR-mediated target gene expressions in the CRPC that could in near future lead to better therapeutic options for patients with CRPC.
